# Poor Outcomes in Patients With Transplant Glomerulopathy Independent of Banff Categorization or Therapeutic Interventions

**DOI:** 10.3389/fmed.2022.889648

**Published:** 2022-05-12

**Authors:** Kaiyin Wu, Danilo Schmidt, Covadonga López del Moral, Bilgin Osmanodja, Nils Lachmann, Fabian Halleck, Mira Choi, Friederike Bachmann, Simon Ronicke, Wiebke Duettmann, Marcel Naik, Eva Schrezenmeier, Birgit Rudolph, Klemens Budde

**Affiliations:** ^1^Department of Nephrology and Intensive Care, Charité-Universitätsmedizin Berlin, Corporate Member of Freie Universität Berlin, Berlin Institute of Health (BIH), Humboldt-Universität zu Berlin, Berlin, Germany; ^2^HLA Laboratory, Charité-Universitätsmedizin Berlin, Corporate Member of Freie Universität Berlin, Humboldt-Universität zu Berlin, BIH, Berlin, Germany; ^3^Institute of Pathology, Charité-Universitätsmedizin Berlin, Corporate Member of Freie Universität Berlin, Berlin Institute of Health (BIH), Humboldt-Universitätzu Berlin, Berlin, Germany

**Keywords:** kidney transplantation, transplant glomerulopathy, chronic antibody-mediated rejection, antihumoral therapy, graft survival

## Abstract

**Background:**

Transplant glomerulopathy (TG) may indicate different disease entities including chronic AMR (antibody-mediated rejection). However, AMR criteria have been frequently changed, and long-term outcomes of allografts with AMR and TG according to Banff 2017 have rarely been investigated.

**Methods:**

282 kidney allograft recipients with biopsy-proven TG were retrospectively investigated and diagnosed according to Banff'17 criteria: chronic AMR (cAMR, *n* = 72), chronic active AMR (cAAMR, *n* = 76) and isolated TG (iTG, *n* = 134). Of which 25/72 (34.7%) patients of cAMR group and 46/76 (60.5%) of cAAMR group were treated with antihumoral therapy (AHT).

**Results:**

Up to 5 years after indication biopsy, no statistically significant differences were detected among iTG, cAMR and cAAMR groups in annual eGFR decline (−3.0 vs. −2.0 vs. −2.8 ml/min/1.73 m^2^ per year), 5-year median eGFR (21.5 vs. 16.0 vs. 20.0 ml/min/1.73 m^2^), 5-year graft survival rates (34.1 vs. 40.6 vs. 31.8%) as well as urinary protein excretion during follow-up. In addition, cAMR and cAAMR patients treated with AHT had similar graft and patient survival rates in comparison with those free of AHT, and similar comparing with iTG group. The TG scores were not associated with 5-year postbiopsy graft failure; whereas the patients with higher scores of chronic allograft scarring (by mm-, ci- and ct-lesions) had significantly lower graft survival rates than those with mild scores. The logistic-regression analysis demonstrated that Banff mm-, ah-, t-, ci-, ct-lesions and the eGFR level at biopsy were associated with 5-year graft failure.

**Conclusions:**

The occurrence of TG is closely associated with graft failure independent of disease categories and TG score, and the long-term clinical outcomes were not influenced by AHT. The Banff lesions indicating progressive scarring might be better suited to predict an unfavorable outcome.

## Introduction

In the last decades it has been recognized, that antibody-mediated rejection (AMR) is an important cause for late allograft failure >1 year after transplantation (1). In our single center AMR was responsible for approximately 1/3 of death-censored allograft losses (2); in a multicenter cohort study AMR caused late allograft dysfunction in up to 60% of renal transplant recipients (3). In clinical reality AMR is frequently a chronic progressive disease process, which starts with the formation of donor specific anti-HLA antibodies (DSA) (4). Next, DSA lead to active AMR in presence of C4d deposition or at least moderate microcirculation inflammation (MVI) (5); over time, TG (defined as Banff cg-lesion) characterized with duplication of the glomerular basement membrane becomes more and more evident, and eventually, results in increasing proteinuria, progressive dysfunction and late allograft loss (6–8). The Banff 2005 report (9) defined chronic active AMR (cAAMR) with three salient features: (i) histological evidence of chronic graft injury (in most cases presence of TG), (ii) the immunohistological evidence of antibody-endothelial interactions by capillary C4d deposition; and (iii) the serological evidence of DSA. Later, a C4d-negative cAAMR was recognized in Banff 2013 report (10), and peritubular capillary C4d deposits could be replaced by at least moderate microcirculation inflammation (MVI). However, it is not uncommon for the three diagnostic features of cAAMR to appear as an incomplete combination, and different features of disease activity in the biopsy may be more reflective of the variable phenotypes of AMR (11). As a consequence, the Banff 2017 report (12) permits the diagnosis of chronic AMR (cAMR) with TG and current or recent DSA in absence of the capillary C4d deposits or at least moderate MVI.

Until recently, some clinical studies have reported that active AMR can be reversed to some degree by a combination of different antihumoral therapies (AHT) including antibody-depletion with plasmapheresis (PPh) or immunoadsorption (IA), immunomodulation with intravenous immunoglobulins (IVIG) with the aid of T- or B-cell-depleting agents (13, 14). The development of strategies to reverse or at least to halt cAAMR remains an unmet medical need, there is no accepted treatment for cAAMR (15, 16). Although TG is the diagnostic hallmark of cAAMR in late stage of transplantation (17), the data on its prognosis and treatment are still limited (18). Moreover, some researches suggest that the presence of TG is relevant to a reduced response to alloantibody removal therapy leading to inevitable late graft failure (19, 20).

According to the most recent Banff criteria (12), cases with TG can be classified into three categories: iTG, cAMR and cAAMR. However, there is no convincing data about the relative impact of these three TG categories on long-term allograft outcomes, partly due to frequent changes in the Banff criteria for AMR since 2001 (21). Thus, more data on cAMR and cAAMR according to the most recent Banff 2017 classification are needed and whether the grading of cg-lesion has any prognostic relevance, which would ease the design of adequate clinical trials to develop effective therapies for the late onset AMR. In addition, only limited data on isolated TG (without the presence for other diseases in the absence of DSA) exist. Therefore, we conducted a single-center retrospective study to investigate the clinical outcomes of allografts with TG with or without AMR according to Banff 2017 criteria and evaluated the prognostic relevance of TG categories and the utility of AHT.

## Materials and Methods

### Study Population and Data Collection

**Figure 1:**
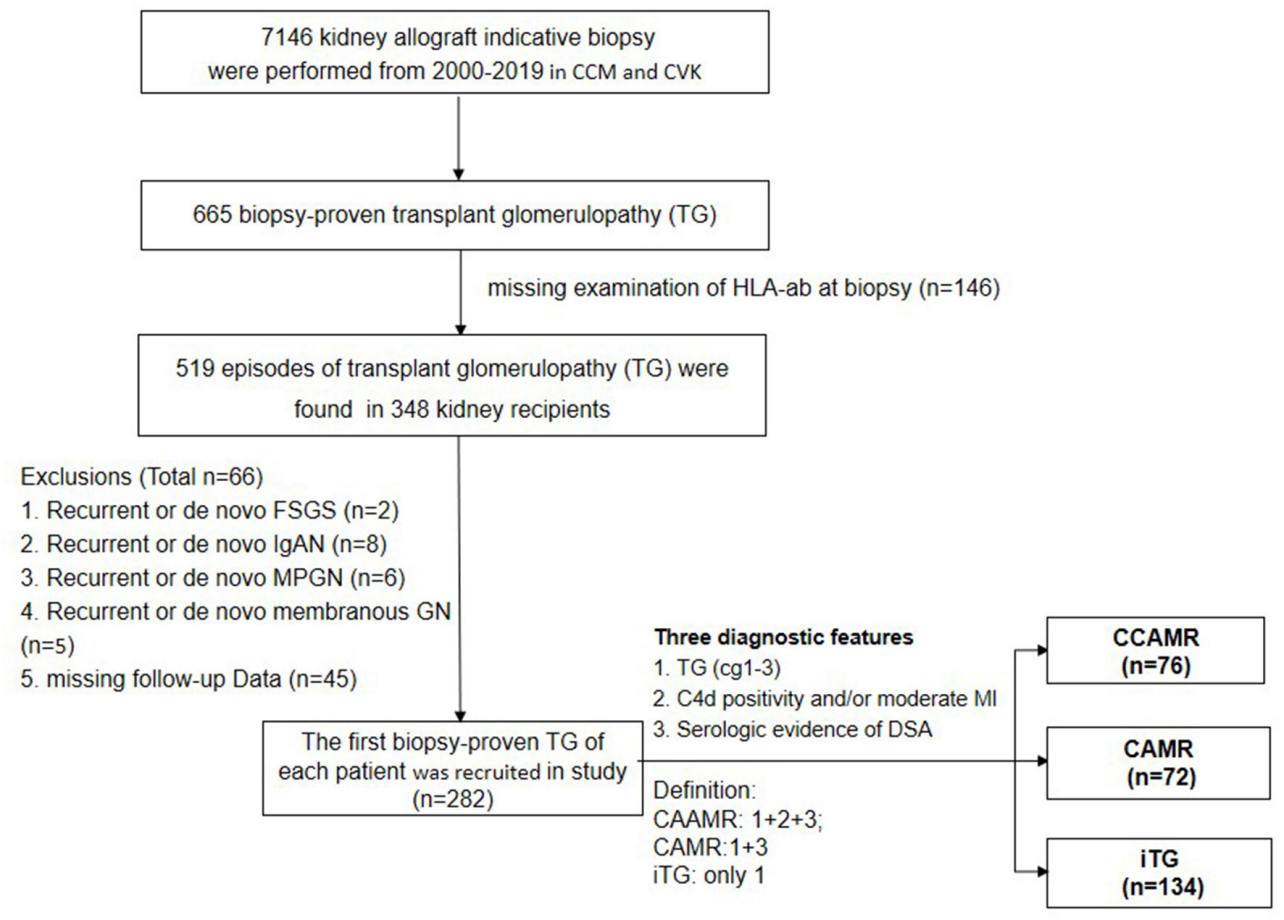
Flow chart of patients enrolled in this study. CCM, Charite Campus Mitte; CVK, Charite Virchow Klinikum. FSGS, focal segmental glomerulosclerosis; IgAN, IgA nephropathy; PGN, membranous proliferative glomerulonephritis; membranos GN, membranous glomerulopathy; iTG, solated transplant glomerulopathy; cAMR, chronic antibody-mediated rejection; cAAMR, chronic active antibody-mediated rejection.

We reviewed all adult patients (≥18 years) who received a single kidney transplantation at the transplant centre of Charité Campus Mitte and Charité Virchow Klinikum. Between Jan, 2000 and Dec, 2019, TG according to Banff 2017 (12) was found in 665 out of 7146 indication biopsies from 494 kidney allograft recipients. 146 patients were excluded because of missing HLA examinations at time of biopsy, 44 patients had incomplete data or were lost to follow-up shortly after biopsy, 21 patients had recurrent or de novo glomerulonephritis; finally, 282 patients with biopsy-proven TG were identified and included into this retrospective study (Figure 1).

All enrolled patients with TG visited routinely the transplantation clinic for follow-up care. The demographic, transplantation characteristics, immunosuppression, and treatment were registered at each outpatient clinic visit in the database (22) and the measurements of eGFR and proteinuria were taken 6 months before and at studied biopsy as well as every 3 months after diagnosis. Database was almost complete with <10% missing values in different data fields. In case of missing values at a certain time point, the next available value was entered. If there were several measurements in one time interval, the measurements at- or nearest to the planned follow-up were entered for analysis. In addition, measurements taken during hospitalization were omitted from analysis to minimize bias due to intercurrent illness and treatment, for example, infection and the admission of intravenous fluids etc.

In order to observe the effects of AHT on the graft outcomes, taking into consideration that in the present study all patients displayed TG, a minimum sample size of 25 patients per group was necessary to detect an eGFR decline difference of 10 ± 10 mL/min/1.73 m^2^ per year between the AHT and free of AHT group (23).

All clinical and laboratory data were selected in the transplant database system (22) and assessed for completeness by a single investigator (S.D). The clinical information was collected from the patients' charts in accordance with the institutional review boards.

### Biopsy and Histopathology

A indication biopsy was performed when the serum creatinine (Scr) rose above 25% from the baseline and/or proteinuria (PU) increased significantly. The biopsy specimens were processed with standard techniques in the institute of pathology, Charité Campus Mitte. All histological slides of recruited biopsies were selected from the archive, reevaluated by two nephropathologists (B.R and K.W) based on the updated Banff classification 2017 (12). TG was distinguished from recurrent or de novo immune complex glomerulopathy by the immunofluorescent and electron microscopy, in particular from membranous/membranoproliferative glomerulonephritis; hepatitis C associated glomerulonephritis and lupus nephritis (24). In addition, TG was separated from thrombotic microangiopathy (TMA) by histological evaluation and review of clinical data (25). TMA was diagnosed based on the presence of typical clinical signs such as coombs negative haemolytic anemia together with thrombocytopenia and one or more of the following histologic conditions (26): fibrin thrombi in glomeruli and/or small arteries and arterioles; endothelial swelling with luminal compromise of the glomerular capillaries; mucoid concentric subintimal thickening of small arteries/arterioles with fragmented and/or hemolyzed erythrocytes; intracapillary or arteriolar thrombosis; vascular fibrinoid necrosis. C4d deposition is detected by indirect immunofluorescence on paraffin sections of formalin-fixed tissue (polyclonal anti-C4d antibody, Dianovo, Germany); more than 1% peritubular capillaries with linear deposition of C4d are considered as positive reaction. The categorization of TG is decided according to Banff report 2017 (12): cAAMR is diagnosed by coexistence of TG, DSA, C4d deposits and /or at least moderate (g+ptc ≥2) MVI; cAMR is considered when both TG and DSA are presented without clues of C4d deposits or at least moderate (g+ptc ≥2) MVI; the cases with TG but in absence of DSA and C4d positivity or with maximal mild MVI (g+ptc <2) are defined as isolated TG. In addition, the isometric vacuolization of proximal tubular epithelium with hyaline vasculopathy, striped pattern interstitial fibrosis and proportional tubular atrophy are considered as calcineurin-inhibitor (CNI) Nephrotoxicity. All Banff lesions are graded on a scale of 0-3 according to the proportion of cortical area affected, with higher scores indicating more severe abnormalities.

### HLA-Antibody Screening

All patients were transplanted with a compliment dependent cytotoxicity (CDC)-negative cross-match. The serum samples at the time of biopsy were evaluated and tested for the presence of donor-specific antibodies against HLA (DSA). If DSA were found to be present, it was determined whether they constituted de novo DSA.

Patient serum samples were collected post biopsy and qualitatively screened for the presence of donor-specific antibodies against HLA (DSA) by two ELISA based screening systems (PRA-STAT and LAT) from 2000 to 2006 and the Luminex assay (27) from 2007 on (Immunocor Transplant Diagnostics Inc., Stamford, CT, USA). Samples that were considered positive for HLA-ab specificities were further analyzed with a Luminex Single Antigen assay (One Lambda, Canoga Park, CA, USA). As an indicator for the antibody level, the maximal fluorescent intensity (MFI) of the immunodominant donor-specific antibody was used. HLA-Ab were considered positive when exceeding a plausible MFI value >500 (28). The values of MFI of immunodominant donor-specific HLA antibodies against class I (panel A) or class II (panel B) antigens were examined at biopsy and during the first year after studied biopsy. All tests were performed according to the manufacturer's guidelines and the DSA level was monitored in regular intervals as previously described (29).

### Immunosuppression and Therapeutic Strategies

The maintennance immunosupression is shown in Table 1. The doses of cyclosporine A (CyA) and tacrolimus (Tac) were adjusted according to whole blood trough levels. 15/72 (20.8%) patients in cAMR group and 16/76 (21.1%) patients in cAAMR group were treated with six sessions PPh (30) followed by intravenous immunoglobulins (IVIG) at 1.5–2.0 g/kg. 6/72 (8.3%) patients in cAMR group and 11/76 (14.5%) patients in cAAMR group received a single dose of rituximab (375 mg/m^2^ body surface area) 1 week after the last IVIG infusion; 4/72 (5.6%) patients in cAMR group and 7/76 (9.2%) patients in cAAMR received bortezomib at 1.3 mg/m^2^ administered intravenously twice weekly on days 1, 4, 8 and 11 after the first IVIG infusion (31). In addition, 6/76 (7.9%) patients in cAAMR group were given 500 mg cyclophosphamide intravenously for 3 rounds after the last IVIG infusion (32) and for 2/76 (2.6%) patients with refractory cAAMR, eculizumab was used as a salvage treatment, a 900-mg dose was repeated weekly until the DSA MFI decreased to 5000. The cases showing concomitant TCMR were given 500 mg methylprednisolone for 3 days and thereafter tapered to maintenance dose at 4 mg/d. After intervention the patients received trimethoprim-sulfamethoxazole as prophylaxis for pneumocystis jirovecii for 6 months. When severe CNI nephrotoxicity (scores of ah, ci and ct-lesion ≥2) was observed, a change in immunosuppression was performed with minimization the doses of CyA/Tac or switch from CNI to a CNI-free immunosuppressive regimen with mTor Inhibitors or belatacept (33).

**Table 1:** Demographics and clinical characteristics.

	**iTG (*n* = 134)**	**cAMR (*n* = 72)**	**cAAMR (*n* = 76)**	**Overall (*n* = 282)**	***P*** **-value**
**Demographics**					
Recipient age (years, median IQR)	40.1 (18–68)	40.5 (18–70)	41.5 (18–78)	40.5 (18–78)	0.91
Recipient gender (m/f)	74/60	35/37	52/24^*^,^#^	161/121	**0.04**
Recipients BMI (kg/m^2^ median, IQR)	25.7 (17.9–36.7)	24.8 (18.3–35.5)	22.8 (19.7–34.4)	24.3 (17.9-36.7)	0.40
First kidney transplant N (%)	113 (84.4%)	57 (79.2%)	64 (84.2%)	234 (83.0%)	0.53
PRA at Tx >10% N (%)	16 (11.9%)	18 (18.1%)	8 (10.5%)	42 (14.9%)	0.34
PRA max before Tx >30% N (%)	23 (17.0%)	18 (18.1%)	10 (13.2%)	52 (18.1%)	0.15
Board HLA-mismatches (N, median IQR)	3.0 (0–6)	2.9 (0–6)	3.2 (0–6)	3.1 (0–6)	0.11
CIT (hours median IQR)	12.1 (0.5–30.5)	5.8 (0.5–28.0)	6.6 (1.0–22.0)	10.0 (0.5–30.5)	0.27
Presence of DGF N (%)	41 (41.4%)	28 (44.4%)	23 (33.3%)	92 (39.8%)	0.39
Donor age (years, median, IQR)	45.3 (3.0-83.0)	49.0 (2.0-94)	48.0 (4.0-80)	48.0 (2.0-94.0)	0.46
Donor gender (m/f)	77/57	34/38	32/44	143/139	0.06
Living donation N (%)	24 (18.2%)	18 (25.0%)	29 (38.2%)^**^	71 (25.4%)	**0.006**
**Clinical characteristics**					
Follow-up after Bx (years, median IQR)	18.3 (1.1–36.3)	15.0 (2.6–29.0)	13.4 (5.0–27.8)	15.9 (1.1–36.3)	0.18
Time of Bx after Bx (years, median IQR)	7.3 (0.3–25.6)	7.1 (0.3–18.7)	6.1 (0.5–20.1)	6.9 (0.3–25.6)	0.13
Follow-up after Bx (years, median IQR)	10.3 (0.6–21.0)	7.6 (0.6-18.5)	6.6 (0.2–14.7)	7.8 (0.2–21.0)	0.54
Time from Bx to detectable DSA (years, median IQR)	–	5.7 (0.0–16.2)	5.0 (0.0–20.1)	5.4 (0.0–20.1)	–
HLA-antibody class type I N(%)	0/134 (0.0%)	12 (16.6%)^**^	12 (15.7%)^**^	24 (8.5%)	**<0.001**
HLA-antibody class type II N(%)	0/134 (0.0%)	52 (72.2%)^**^	36 (47.4%)^**^, ^##^	88 (31.2%)	**<0.001**
HLA-antibody class type I+II N(%)	0/134 (0.0%)	8 (11.1%)^*^	28 (36.8%)^**^, ^##^	36 (12.8%)	**<0.001**
**Maintenance immunosuppression regimens at Bx N (%)**					
Tac+MMF/MPA+PDN	50 (37.0 %)	42 (58.3 %)	39 (53.4 %)	131 (46.5%)	0.58
CyA+MMF/MPA+PDN	38 (28.1 %)	18 (25.0 %)	17 (23.3 %)	73 (25.9%)	0.49
Rap+MMF/MPA+PDN	4 (3.0 %)	1 (1.4 %)	4 (5.5 %)	9 (3.2%)	0.70
Tac+MMF/MPA	4 (3.0 %)	2 (2.8 %)	5 (6.8 %)	11 (3.9%)	0.45
CyA+MMF/MPA	10 (7.4 %)	3 (4.2 %)	4 (5.5 %)	17 (6.0%)	0.66
CyA+Azathioprine+PDN	21 (15.6 %)	4 (5.6 %)	1 (1.4 %)	26 (7.3%)	0.08
Tac+PDN	3 (2.2 %)	1 (1.4 %)	1 (1.4 %)	5 (1.8%)	0.81
CyA+PDN	3 (2.2 %)	0 (0.0 %)	1 (1.4 %)	4 (1.4%)	0.78
MMF/MPA+PDN	2 (1.5 %)	1 (1.4 %)	1 (1.4 %)	4 (46.5%)	0.93
**(D) Antihumoral treatment (AHT) N (%)**					
PPh+IVIG	0/134 (0.0%)	15 (20.8 %)^**^	16 (21.1 %)	31(11.0%)	**<0.001**
PPh+IVIG+rituximab^+^	0/134 (0.0%)	6 (8.3 %)	11 (14.5 %)	17 (6.0%)	**<0.001**
PPh+IVIG+bortezomib^+^	0/134 (0.0%)	4 (5.6 %)^*^	7 (9.2 %)^*^, ^#^	11 (3.9%)	**0.01**
PPh+IVIG+cyclophosphamide^+^	0/134 (0.0%)	0 (0.0 %)	6 (7.9 %)^*^, ^#^	6 (2.1%)	**0.03**
PPh+IVIG+eculizumab^+^	0/134 (0.0%)	0 (0.0 %)	2 (2.6 %)	2 (0.7%)	0.08
Patients receiving AHT^+^	0/134 (0.0%)	25 (34.7 %)^**^	42 (55,3 %)^**^, ^##^	67 (23.8 %)	**<0.001**
Steroid bolus	13 (9.6 %)	9 (12.5 %)	30 (40.0 %)^**^, ^##^	52 (18.4%)	**<0.001**
**(E) Presence of adverse events in the 12 months post Bx**					
Urinary tract infection N (median IQR)	0.2 (0–5)	0.3 (0–5)	0.4 (0–5)	0.3 (0–5)	0.12
Respiratory tract infection N (median IQR)	0.3 (0–1)	0.2 (0–1)	0.3 (0–1)	0.3 (0–1)	0.18
CMV infectious colitis N (median IQR)	0.1 (0–1)	0.1 (0–2)	0.1 (0–1)	0.1 (0–2)	0.33
Polyoma virus nephropathy N (median IQR)	0.2 (0–2)	0.1 (0–1)	0.1 (0–1)	0.1 (0–2)	0.19
**(F) The level of HbA1c and blood pressure at Bx**					
HbA1c level (%median IQR)	5.3 (4.7–7.2)	5.4 (4.6–7.7)	5.2 (4.3–7.5)	5.3 (4.6–7.7)	0.75
SBP level (mmHg median IQR)	140 (100–221)	140 (110–204)	139 (72–180)	140 (72–221)	0.83
DBP level (mmHg median IQR)	84 (55–119)	80 (60–110)	80 (60–101)	82 (55–119)	0.92
**(G) Antihypertensive therapy after Bx**					
ACEi N (%)	34 (25.2 %)	20 (27.8 %)	23 (30.3 %)	77 (27.3%)	0.43
ARB N (%)	29 (21.3 %)	12 (16.7 %)	25 (32.9%)	66 (23.4%)	0.56
CCB N (%)	39 (28.9 %)	18 (13.3 %)	32 (42.1 %)	89 (31.6%)	0.58
Beta-blocker N (%)	8 (5.9 %)	4 (5.6 %)	9 (11.8 %)	21 (7.4%)	0.52

*IQR, interquartile range; BMI, body mass index; ESRD, end stage renal disease; CIT, cold ischemic time*.

*PRA, panel reactive antibody; HLA, human leukocyte antigen; DSA, donor-specific anti-HLA antibodies*.

*Bx, the studied biopsies; MMF, mycophenolate mofetil; MPA, mycophenolic acid; Tac, Tacrolimus; CyA, Cyclosporin A; Rap, rapamycin; PDN, Predinisolon; ACEi, angiotensin converting enzyme inhibitors; ARB, angiotensin receptor blocker; CCB, calcium canal antagonist; MFI, mean fluorescent intensity; HbA1c, hemoglobin A1c; SBP, systolic blood pressure; DBP, diastolic blood pressure*.

** *p < 0.01, comparing with iTG;*

* *p < 0.05, comparing with iTG*.

## *p < 0.01, comparing with cAMR;*

# *p < 0.05, comparing with cAMR*.

*The bold values indicates all p-values less than 0.05*.

In addition, patients with hypertension received at least one antihypertensive drug and patients with daily urinary protein excretion (e.g., >1 g/L) were treated with the maximum tolerable dose of an angiotensin converting enzyme inhibitors (ACEi) and/or angiotensin receptor blockers (ARB) with the aid of AHT in patients of cAMR and cAAMR group.

### Clinical Outcomes

All patients were followed up until the end of our study on 31.12.2020 or irreversible return to the chronic dialysis or retransplantation. Change in renal allograft function in time was evaluated by estimated glomerular filtration rate (eGFR ml/min/1.73 m^2^) and urinary protein excretion. The eGFR value was calculated using formula of the Modification of Diet in Renal Disease (MDRD) (34). The influence of TG categories and AHT on eGFR slope was evaluated using linear mixed models with eGFR levels from 0, 12, 24, 36, 48, and 60 months postbiopsy as dependent variables, the interaction of TG categories or AHT and time as fixed effects. The covariance structure was specified as an autoregressive model of the first order. In model A patients experiencing graft loss or after death, the value of eGFR was not imputed. For an additional sensitivity analyses (model B), eGFR after graft loss or death was set to 5 ml/min/1.73 m^2^. The effect of TG categories and AHT on long-term outcome was analyzed for patient and graft survival over a 5-year period after indication biopsy.

### Statistical Analysis

Continuous data were expressed as median (IQR) and categorical variables were expressed as N and percentage of total. Mann-Whitney U test was used for comparison of continuous variables and chi-square for categorical data. The calculation of patient- and graft survival was analyzed by Kaplan-Meier curves and log-rank test. For univariate analysis of the histological factors influencing the 5-year death-censored graft failure we performed a Kaplan-Meier analysis for each histological Banff lesion comparing mild (score 0-1) and severe (score 2-3) lesion scores. The Log Rank test was used for statistical comparison between cases with mild and severe grade of each Banff lesion, and the Banff lesions with *p*-values < 0.05 were selected for further multivariable analysis. For multivariable modeling, a binary-logistic regression analysis was employed to examine the effects of three selected clinical factors (receiving antihumoral therapy, eGFR and proteinuria at biopsy) on overall graft survival, patient survival and death-censored graft survival. Adjusted estimates from multivariable models are presented as odds ratios (OR) with 95% confidence intervals (CI). All statistics were performed by using SPSS16.0 (SPSS Inc., Chicago, IL), *P*-value < 0.05 was considered as significant.

## Results

### Clinical Characteristics at Studied Biopsy

In total, 282 patients with first episode of biopsy-proven TG and complete follow-up were enrolled in this study and were reclassified into cAMR (*n* = 72), cAAMR (*n* = 76) and iTG (*n* = 134) groups. Moreover, 25/72 (34.7%) patients in cAMR group and 46/76 (60.5%) patients in cAAMR group were treated with AHT primarily consisting of high-dose IVIG and PPh (Table 1). The basic demographics (including age, sex, body mass index) as well as transplant characteristics (including the presence of DGF, HLA-mismatches, PRA max before and at transplantation, type of donation) are summarized in Table 1. Baseline characteristics did not differ significantly among three groups with exception of significantly more male recipients and living donors in cAAMR group in comparison with iTG and cAMR group as well as the evidently higher fraction of living donation in cAAMR group vs. iTG group.

### Transplant Characteristics at Transplantation and Studied Biopsy

TG was first diagnosed at a median of 6.9 (0.3–25.6) years without notable differences among iTG, cAMR and cAAMR groups and similar follow-up (Table 1). DSA were detected at a median time of 5,7 years post transplantation in cAMR group and 5.0 years in cAAMR group (*P* = 0.89).

In the cAMR group, 12/72 (16.7%) patients had only class I HLA-antibodies vs. 12/76 (15.8%) patients of cAAMR group (*P* = 0.91). In 52/72 (72.2%) patients of cAMR group and 35/76 (46.1%) patients of cAAMR group, only class II HLA-antibodies were detected (*P* = 0.002), and in 8/72 (11.1%) patients of cAMR group and 28/76 (36.8%) patients of cAAMR group, both class I and II HLA-antibodies (*P* < 0.001). The vast majority of DSA were found to be de novo DSA (93.1% of cAMR group vs. 97.4% of cAAMR group, *P* = 0.79) and patients with cAMR had a predominance of only class II DSA, while cAAMR had more frequently class I and II DSAs. The median value of the immunodominant DSA (MFI_max) tended to be higher in cAAMR group than in cAMR group without reaching the significantly different level.

No significant differences of the distribution of the maintenance immunosuppression regimens and ACE inhibitors or ARBs were found among iTG, cAMR and cAAMR groups. The AHT regimens were given with the comparable fraction to the patients in cAMR and cAAMR groups (Table 1).

### Histological Evaluation of the Studied Biopsies

The detailed biopsy diagnoses and kidney pathology lesion scores are shown in Table 2. A median of 12 glomeruli (1, 7–49) was available per biopsy, a median of 13% (0–80%) glomeruli presented with global sclerosis; no significant differences were found for the number of detectable glomeruli (*P* = 0.05) and the percentage of glomerulosclerosis (*P* = 0.79) among iTG, cAMR and cAAMR groups. The median g-, ptc- and cg-lesion scores in cAAMR group were significantly higher than those in iTG and cAMR group (each comparison: *P* < 0.001). A concomitant TCMR including borderline rejection was found in 13/134 (9.6%) patients of iTG group and 9/72 (12.5%) patients of cAMR group vs. 30/76 (40.0%) patients of cAAMR group (*P* < 0.001). Furthermore, among three TG groups no significant differences were found with respect to the chronic interstitial fibrosis/ tubular atrophy (by ci- and ct-lesion) or chronic vascular change (by ah- and cv-lesion).

**Table 2:** Morphologic results of studied biopsies and of 60-month follow-up.

	**iTG (*n* = 134)**	**cAMR (*n* = 72)**	**cAAMR (*n* = 76)**	**Overall (*n* = 282)**	***P*** **-value**
Total detected glomeruli N (IQR)	11 (7–53)	12 (7–50)	13 (7–51)	12 (7–53)	0.05
Global glomerulosclerosis % (IQR)	13 (0–80)	14 (0–75)	16 (0–65)	13 (0–80)	0.89
Total interlobular arteries N (IQR)	1.5 (1–7)	1.5 (1–4)	1.5 (1–8)	1.5 (1–8)	0.79
**Histological scores of Banff-lesions at Bx (scores median IQR)**					
g (0–3)	0.1 (0–1)	0.1 (0–3)	1.8 (0–3)^**^, ^##^	0.4 (0–3)	**<0.001**
ptc (0–3)	0.1 (0–3)	0.1 (0–3)	1.6 (0–3)^**^, ^##^	0.3 (0–3)	**<0.001**
cg (0–3)	2.0 (1–3)	2.2 (1–3)	2.6 (1–3)^*^, ^#^	1.0 (1–3)	**<0.001**
C4d (0–3)	0.0 (0–0)	0.0 (0–0)	0.6 (0–3)^**^, ^##^	0.2 (0–3)	**<0.001**
v (0–3)	0.0 (0–2)	0.1 (0–2)	0.2 (0–3)	0.1 (0–3)	**0.03**
ci (0–3)	1.0 (0–3)	0.7 (0–3)	0.9 (0–3)	0.9 (0-3)	0.13
ct (0–3)	1.0 (0–3)	0.7 (0–3)	0.9 (0–3)	0.9 (0-3)	0.15
i (0–3)	0.8 (0–3)	0.7 (0–3)	1.0 (0–3)	0.8 (0–3)	0.26
mm (0–3)	1.1 (0–3)	0.9 (0–3)	0.9 (0–3)	1.0 (0–3)	0.28
ah (0–3)	2.4 (0–3)	2.4 (0–3)	2.5 (0–3)	2.4 (0–3)	0.61
t (0–3)	0.3 (0–3)	0.4 (0–3)	0.4 (0–3)	0.4 (0–3)	0.79
cv (0–3)	1.8 (0–3)	1.9 (0–3)	1.9 (0–3)	1.8 (0–3)	0.86
At least moderate MVI N (%)	0 (0.0%)	0 (0.0%)	69 (90.8%)^**^, ^##^	69 (24.5%)	**<0.001**
Advanced IFTA (ci3+ct3) N (%)	7 (5.3%)	4 (5.6%)	10 (13.2%)	21 (7.5%)	0.15
Concomitant TCMR N (%)	13 (9.6 %)	9 (12.5 %)	30 (40.0 %)^**^, ^##^	52 (18.4%)	**<0.001**
**Histological diagnosis of indication biopsies during 60-month postbiopsy follow-up**					
≥1 for-cause Bx after studied Bx N (%)	54/134 (40.3%)	29/72 (40.3%)	44/76 (57.9%)^*^, ^#^	127/282 (45.0%)	**0.02**
≥1 episode of iTG, N (%)	35/54 (64.8%)	0/29 (0.0 %)^**^	0/44 (0.0 %)^**^	35/127 (27.6%)	**<0.001**
≥1 episode of cAMR, N (%)	9/54 (16.7 %)	21/29 (72.4 %)^**^	12/44 (27.3 %)^##^	42/127 (33.1%)	**<0.001**
≥1 episode of cAAMR, N (%)	1/54 (0.7 %)	10/29 (34.5 %)	33/44 (75.0 %)^**^, ^##^	44/127 (34.6%)	**<0.001**
≥1 episode of advanced IFTA (ci3+ct3), N (%)	13/54 (24.1%)	3/29 (10.3%)	1/44 (2.3%)	17/127 (13.4%)	**0.04**

*Banff scored lesions: glomerulitis (g); peritubular capillaritis (ptc); transplant glomerulopathy (cg); intimal arteritis (v); interstitial inflammation (i); tubulitis (t); mesangial matrix increase (mm); vascular intimal thickening (cv); arteriolar hyaline thickening (ah); interstitial fibrosis (ci) and tubular atrophy (ct); at least moderate MVI: g+ptc ≥2; advanced IFTA: ci3+ct3; Bx, the studied biopsies; concomnitant TCMR, co-existed borderline rejection and Banff TCMR typies*.

** *p < 0.01, comparing with iTG;*

* *p < 0.05, comparing with iTG*.

## *p < 0.01, comparing with cAMR;*

# *p < 0.05, comparing with cAMR*.

*The bold values indicates all p-values less than 0.05*.

During follow-up, a total of 190 indication biopsies in 127 patients (54, 29 and 44 patients in iTG, cAMR and cAAMR groups, respectively) were performed. Most biopsies confirmed the previous diagnosis and only a few patients changed categories (Table 2). Moreover, the advanced IFTA characterized with highest score of ci and ct (ci3+ct3) was found in 13/54 (24.1%) patient of iTG group, 3/29 (10.3%) patients of cAMR group and 1/44 (2.3%) patient of cAAMR group (*P* = 0.04).

### Effect of TG Categories on the Kidney Allograft Function

**Figure 2:**
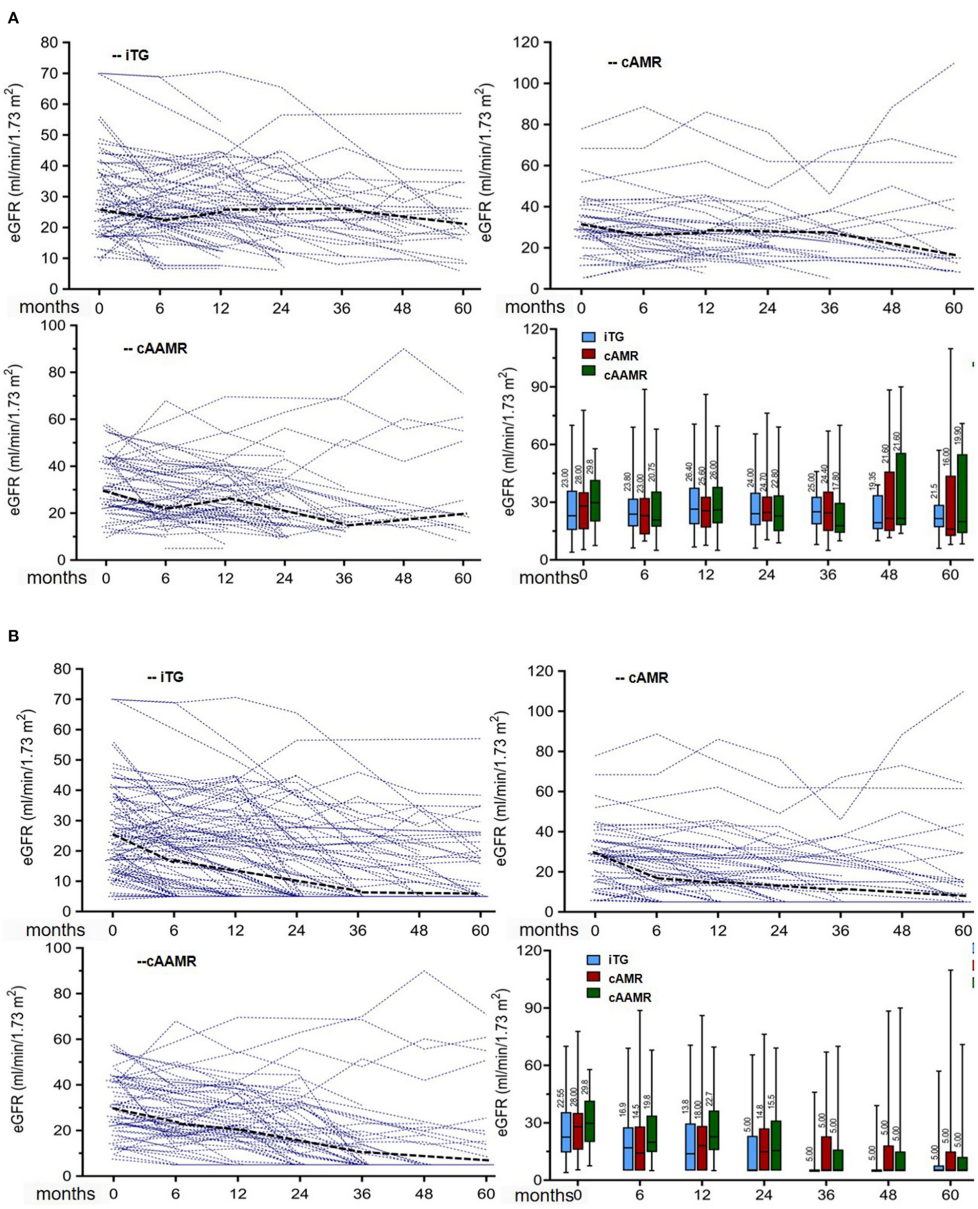
**(A)** Effects of TG categories on the evolution of eGFR, for patient death or return to dialysis, no data of eGFR were imputed. **(B)** Effects of TG categories on the evolution of eGFR, for patient return to dialysis, eGFR were imputed as 5 ml/min/1.73 m^2.^ Individual eGFR course (thin dashed lines) and median eGFR (fat dashed lines) in relation to TG categories. Analyses are on the basis of serial eGFR measurements at 0, 6, 12, 18, 24, 36, 48, and 60 months. Box plots indicate the median, the interquartile range, the minimum, and the maximum of the measures.

The median eGFR (Table 3A) 6 months before biopsy was 28.2 ml/min/1.73 m^2^ in iTG group and 33.3 ml/min/1.73 m^2^ in cAMR group, which were significantly lower than 41.5 ml/min/1.73 m^2^ in cAAMR group (*P* = 0.03). After biopsy, most patients had a progressive decline in renal function and median eGFR during follow up was similar among groups (each comparison among three groups at time post biopsy *P* > 0.05) without imputation (model A Figure 2A) and with imputation of graft loss (model B Figure 2B).

**Table 3A:** Variation of DSA, estimated glomerular filtration rate (eGFR), and daily proteinuria pre-, at-, and post-studied biopsies in relation to TG categories.

	**iTG (*n* = 134)**	**cAMR (*n* = 72)**	**cAAMR (*n* = 76)**	**Overall (*n* = 282)**	***p*** **–value**
**DSA–MFI intensicy at and after Bx (median IQR)**					
MFI_max at Bx (median IQR)	–	5071 (380–23137)	9758 (327–22438)	8701 (327–23137)	0.15
MFI_max at 6 months post Bx (median IQR)	–	5109 (528–25113)	10688 (330–23320)	9310 (330–25113)	0.08
MFI_max at 1 year post Bx (median IQR)	–	5018 (343–23302)	13012 (397–26436)	9039 (343–26436)	0.05
**Model A: The eGFR values before and after Bx (ml/min/1.73 m**^**2**^ **median IQR)**					
eGFR 6 months before Bx	28.2 (15.3–66.1)	33.3 (10.5–88.6)	41.5 (11.9–83.6)^**^	35.9 (7.7–88.6)	**0.03**
eGFR at Bx	24.7 (4.0–70.0)	28.0 (5.4–77.8)	29.8 (7.5–57.8)	26.0 (4.0–77.8)	0.26
eGFR 6 months after Bx	23.8 (6.2–69.0)	23.0 (9.8–88.7)	21.0 (9.8–68.0)	22.4 (6.2–88.7)	0.91
eGFR 1 year after Bx	26.4 (6.7–70.6)	25.6 (7.6–86.1)	26.0 (6.6–69.6)	26.2 (4.0–139.0)	0.96
eGFR3 years after Bx	25.0 (8.0–46.0)	24.4 (5.0–67.0)	17.8 (9.9–70.0)	22.5 (5.0–70.0)	0.99
eGFR 5 years after Bx	21.5 (6.0–57.0)	16.0 (5.0–51.0)	20.0 (10.0–70.9)	20.0 (5.0–70.9)	0.43
**Model A: The decline of eGFR at and after the studied Bx (ml/min/1.73 m**^**2**^ **median IQR)**					
Δ eGFR 6 months before Bx	−3.1 (−19.8–13.5)	−5.1 (−57.3–6.0)^*^	−11.0 (−53.8–32.5)^**^	−6.5 (−57.3–32.5)	**0.02**
Δ eGFR Bx to 6 months after Bx	−2.9 (−32.2–19.9)	−1.4 (−17.4–10.9)	−5.2 (−32.0–27.3)	−3.4 (−32.2–27.3)	0.58
Δ eGFR Bx to 1 year after Bx	−4.3 (−32.2–15.6)	−5.8 (−18.7–17.7)	−4.8 (−28.4–21.4)	−5.0 (−32.2–21.4)	0.92
Δ eGFR Bx to 3 years after Bx	−6.4 (−23.5–12.1)	−9.8 (−22.4–15.0)	−9.0 (−48.1–30.7)	−8.7 (−48.1–30.7)	0.45
Δ eGFR Bx to 5 years after Bx	−6.3 (−52.3–16.0)	−9.5 (−43.0–31.6)	−5.7 (−27.2–30.2)	−6.3 (−52.3–31.6)	0.60
**Model B: The eGFR values before and after Bx (ml/min/1.73 m**^**2**^ **median IQR)**					
eGFR 6 months before Bx	28.2 (15.3–66.1)	33.3 (10.5–88.6)	41.5(11.9–83.6)^**^	35.9 (7.7–88.6)	**0.03**
eGFR at Bx	24.7 (4.0–70.0)	28.1 (5.4–77.8)	29.8 (7.5–57.8)	25.8 (4.0–77.8)	0.16
eGFR 6 months after Bx	16.9 (5.0–69.0)	17.5 (5.0–86.1)	22.7 (4.0–139.0)	16.2 (5.0–88.7)	0.26
eGFR 1 year after Bx	13.8 (5.0–70.6)	14.8 (5.0–76.3)	19.8 (5.0–68.0)	17.0 (4.0–139.0)	0.05
eGFR3 years after Bx	5.8 (5.0–46.0)	11.1 (5.0–67.0)	8.3 (5.0–70.0)	8.9 (5.0–70.0)	0.10
eGFR 5 years after Bx	5.4 (5.0–57.0)	7.1 (5.0–51.0)	6.3 (5.0–70.9)	6.5 (5.0–70.9)	0.12
**Model B: The decline of eGFR at and after Bx (ml/min/1.73m**^**2**^ **median IQR)**					
Δ eGFR 6 months before Bx	−3.1 (−19.8–13.5)	−5.1 (−57.3–6.0)^*^	−11.0 (−53.8–32.5)^**^	−6.5 (−57.3–32.5)	**0.02**
Δ eGFR Bx to 6 months after Bx	−3.0 (−32.2–19.9)	−2.7 (−17.4–10.9)	−4.9 (−32.0–27.3)	−3.9 (−32.2–27.3)	0.85
Δ eGFR Bx to 1 year after Bx	−5.5 (−34.0–15.6)	−6.1 (−23.9–17.7)	−5.0 (−28.4–21.4)	−5.6 (−34.0–21.4)	0.61
Δ eGFR Bx to 3 years after Bx	−12.0 (−70.0–11.7)	−11.9 (−37.6–15.0)	−15.0 (−50.2–29.3)	−12.1 (−70.0–29.3)	0.48
Δ eGFR Bx to 5 years after Bx	−12.1 (−65.0–16.0)	−11.4 (−43.0–41.4)	−19.7 (−50.2–30.2)	−13.0 (−65.0–41.4)	0.49
**The proteinuria excretion before and after Bx (mg/day median IQR)**					
PU 6 months before Bx	896 (39–6758)	709(40–5312)	866 (67–12181)	835 (39–12181)	0.54
PU at Bx	1474 (54–6962)	1271 (87–8366)	955 (90–6540)	1081(54–8366)	0.30
PU 6 months after Bx	1040 (93–5807)	1019(48–11597)	1062 (65–9886)	1040 (48–11597)	0.73
PU 1 year after Bx	810 (82–5373)	871 (82–5074)	934 (66–6605)	869 (82–6605)	0.74
PU 3 years after Bx	645 (171–1681)	998 (67–5204)	667 (176–3186)	725 (67–5204)	0.32
PU 5 years after Bx	496 (94–7688)	949 (94–2459)	761 (73–4078)	629 (73–7688)	0.87
**The variation of proteinuria at and after Bx (mg/day median IQR)**					
Δ PU 6 months before Bx	163 (−3454–5744)	−42 (−4465–3187)	116 (−3722–5050)	121 (−4465–5744)	0.09
Δ PU Bx to 6 months after Bx	−125 (−5578–8732)	44 (−950–8201)	123 (−1920–6079)	−5 (−5578–8734)	0.07
Δ PU Bx to 1 year after Bx	−143 (−5781–3099)	40 (−3619–1577)	77 (−2184–7430)	−16 (−5781–7430)	0.06
Δ PU Bx to 3 years after Bx	132 (−5308–2434)	480 (−3000–3420)	48 (−1713–3211)	106 (−5308–3420)	0.21
Δ PU Bx to 5 years after Bx	146 (−2606–2429)	885 (−1566–1232)	272 (−653–1891)	147 (−2606–2429)	0.85

*The values were expressed as median and IQR*.

*MFI, mean fluorescent intensity; eGFR, estimated glomerular filtration rate; ΔeGFR, difference of eGFR value*.

*PU, daily urine protein excretion; ΔPU, difference of PU value*.

*Model A, the eGFR values after graft loss or death were not imputed*.

*Model B, the eGFR values after graft loss or death was imputed as 5 ml/min/1.73 m^2^*.

** *p < 0.01, comparing with iTG;*

* *p < 0.05, comparing with iTG*.

*The bold values indicates all p-values less than 0.05*.

The evolution of eGFR is analyzed by linear mixed model and illustrated in Supplementary Table 1 with and without imputation for graft loss or death, which did not reveal any significant difference of TG categories in association with eGFR decline (*F* = 1.3, *P* = 0.28), and there was no statistical difference in eGFR decline among three groups. The mean annual eGFR decline of iTG, cAMR and cAAMR group were −3.2 (95%CI, −5.2 to −1.2), −2.5 (95%CI, −4.5 to 0.5) and −2.9 (95%CI, −4.9 to 0.9) ml/min/1.73m^2^/year, respectively (each comparison: *P* > 0.05). The difference of annual eGFR decline (ml/min/1.73 m^2^ per year) was not significant when the comparison was performed between each two TG categories. In addition, there was no significant difference in proteinuria pre-, at- and post-diagnosis among iTG, cAMR and cAAMR groups (comparison in each time yields *P* > 0.05).

### Effect of AHT on Renal Allograft Function

In order to analyze the effect of AHT on DSA intensity and allograft function, cAMR and cAAMR groups were further divided into subgroups based on AHT. As shown in Table 3B, no significant differences were found with regard to DSA intensity, allograft function and proteinuria, and no statistical significances were found in comparison to untreated iTG group. The association of AHT with eGFR slope was analyzed by linear mixed model and is shown in Supplementary Table 2. The difference of annual eGFR decline (ml/min/1.73 m^2^/year) was not significant when the comparison was performed between different groups with and without treatment, irrespective of imputation. Similarly, proteinuria was similar in all groups during follow-up (*P* > 0.05).

**Table 3B:** Variation of DSA, estimated glomerular filtration rate (eGFR), and proteinuria in relation to antihumoral therapy.

	**iTG** ** (*n* = 134)**	**cAMR free of AHT** ** (*n* = 47)**	**cAMR with AHT ** **(*n* = 25)**	**cAAMR free of AHT** ** (*n* = 30)**	**cAAMR with AHT** ** (*n* = 46)**	***p*** **-value**
**DSA-MFI intensity at and after Bx (median IQR)**						
MFI_max at Bx	–	8046 (380–23137)	4273 (648–25113)	11782 (386–22277)	8941 (327–22438)	0.12
MFI_max at 6 months after Bx	–	8798 (320–21105)	3984 (405–22516)	11153 (1863–18265)	10001 (330–23320)	0.91
MFI_max at 1 year after Bx	–	6913 (420–23076)	3606 (343–23302)	9321 (1505–22460)	14784 (397–26436)	0.05
**Model A: The eGFR values before and after Bx (ml/min/1.73 m**^**2**^ **median IQR)**						
eGFR 6 months before Bx	28.2 (15.3–66.1)	29.0 (18.9–81.8)	41.5 (10.5–88.6)	36.0 (15.0–72.2)	42.0 (11.9–83.6)^**^	0.06
eGFR at Bx	24.7 (4.0–70.0)	28.3 (5.4–77.8)	27.7 (5.4–52.0)	26.3 (9.1–57.8)	30.5 (7.5–57.4)	0.54
eGFR 6 months after Bx	23.8 (6.2–69.0)	26.2 (11.6–88.7)	14.9 (9.8–43.8)	22.5 (10.8–48.9)	19.8 (9.8–68.0)	0.52
eGFR 1 year after Bx	26.4 (6.7–70.6)	26.6 (13.2–86.1)	19.7(7.6–62.2)	26.0 (12.1–69.6)	26.8(6.6–54.4)	0.84
eGFR3 years after Bx	25.0 (8.0–46.0)	20.5 (5.0–46.0)	25.9 (14.0–67.0)	16.0 (15.0–68.6)	18.0 (9.9–70.0)	0.78
eGFR 5 years after Bx	21.5 (6.0–57.0)	15.5 (8.0–109.8)	29.5 (8.8–64.0)	12.8 (8.3–60.9)	25.5 (15.3–70.9)	0.63
**Model A: The decline of eGFR at and after Bx (ml/min/1.73 m**^**2**^ **median IQR)**						
Δ eGFR 6 months before Bx	−3.1 (−19.8–13.5)	−2.4 (−13.5–6.0)	−12.5 (−57.3– −0.5)^*^	−15.2 (−24.5– −0.9)^*^, ^#^	−11.1 (−53.8–32.5)^**^	0.005
Δ eGFR 6 months after Bx	−3.4 (−22–16.7)	−1.8 (−18.8–25.0)	−4.3 (−18.8–7.0)	−2.3 (−26.7–22.3)	−2.4 (−14.6–38.4)	0.58
Δ eGFR 1 year after Bx	−4.1 (−24.1–15.6)	−4.7 (−17.0–21.1)	−7.5 (−24.8–15.0)	−6.5 (−37.2–13.3)	−3.7 (−27.0–21.4)	0.13
Δ eGFR 3 years after Bx	−6.4 (−23.5–12.1)	−13.0 (−22.4–9.4)	−3.5 (−20.0–15.0)	−9.7 (−28.3–20.4)	−8.1 (−48.1–30.7)	0.28
Δ eGFR 5 years after Bx	−6.3 (−52.3–16.0)	−15.9 (−43.0–31.6)	−3.5 (−17.0–8.0)	−8.3 (−27.2–12.7)	−5.6 (−26.2–30.2)	0.62
**Model B: The eGFR values before and after the studied Bx (ml/min/1.73 m**^**2**^ **median IQR)**						
eGFR 6 months before Bx	28.2 (15.3–66.1)	29.0 (18.9–81.8)	41.5 (10.5–88.6)	36.0 (15.0–72.2)	42.0 (11.9–83.6)^**^	0.06
eGFR at Bx	24.7 (4.0–70.0)	28.3 (5.4–77.8)	27.7 (5.4–52.0)	26.3 (9.1–57.8)	30.5 (7.5–57.4)	0.39
eGFR 6 months after Bx	16.9 (5.0–69.0)	19.1 (5.0-88.7)	12.2 (5.0–43.8)	20.8 (5.0–48.9)	19.7 (5.0–68.0)	0.43
eGFR 1 year after Bx	13.8(5.0–70.6)	19.0 (5.0–86.1)	16.8 (5.0–62.2)	21.1 (5.0–69.6)	23.2 (5.0–54.4)	0.19
eGFR3 years after Bx	5.8 (5.0–46.0)	12.4 (5.0–46.0)	9.9 (5.0–67.0)	7.5 (5.0–68.6)	9.4 (5.0–70.0)	0.25
eGFR 5 years after Bx	5.4 (5.0–57.0)	8.0 (5.0–109.8)	6.6 (5.0–64.0)	6.3 (5.0–60.9)	8.8 (5.0–70.9)	0.22
**Model B: The decline of eGFR at and after Bx (ml/min/1.73 m**^**2**^ **median IQR)**						
Δ eGFR 6 months before Bx	−3.1(−19.8–13.5)	−2.4 (−13.5–6.0)	−12.5 (−57.3–−0.5)^*^	−15.2 (−24.5– −0.9)^*^^, ^Δ^^	−11.1 (-53.8–32.5)^**^	0.005
Δ eGFR 6 months after Bx	−3.0 (−32.2–19.9)	−1.7 (−16.3–10.9)	−4.5(−17.4–7.6)	−4.7 (−18.0–13.0)	−4.9 (−32.0–27.3)	0.98
Δ eGFR 1 year after Bx	−5.4 (−34.0–15.6)	−6.7 (−17.0–17.7)	−5.7 (−23.9–15.0)	−3.9 (−18.4–21.4)	−5.4 (−28.4–18.3)	0.78
Δ eGFR 3 years after Bx	−12.0 (−70.0–11.7)	−13.0 (−24.7–8.6)	−9.0 (−37.6–15.0)	−17.9 (−50.2–20.4)	−13.0 (−49.6–29.3)	0.75
Δ eGFR 5 years after Bx	−12.1 (−65.0–16.0)	−13.2 (−43.0–41.4)	−7.7 (−37.6–12.0)	−22.6 (−50.2–12.7)	−18.9 (−42.1–30.2)	0.66
**The proteinuria values at and after Bx (mg/day median IQR)**						
PU 6 months before Bx	896 (39–6758)	991(59–5155)	653 (45–2613)	866 (67–12181)	955 (90–6540)	0.54
PU at Bx	1474 (54–6962)	918 (48–11579)	969 (143–5812)	852 (78–4563)	1061.5 (65–9886)	0.48
PU 6 months after Bx	1040 (9–5807)	665(89–6989)	1114 (208–3732)	1058(59–6605)	998 (41–12355)	0.19
PU 1 year after Bx	684 (84–3812)	1037 (137–3325)	462 (125–3732)	165 (60–2637)	800 (41–12355)	0.58
PU 3 years after Bx	766 (75–4661)	1656 (75–3420)	841 (445–2600)	613 (184–1042)	540 (203–3172)	0.43
PU 5 years after Bx	539 (50–3581)	1365 (50–2206)	909 (199–1641)	622 (107–1818)	629 (158–2,404)	0.88
**The variation of proteinuria at and after Bx (mg/day median IQR)**						
Δ PU 6 months before Bx	163 (−3,454–5,744)	−21 (−4,465–1,598)	−65 (−950–3,187)	132 (−3732–5,050)	84 (−2,066–4,057)	0.11
Δ PU 6 months after Bx	−125 (−5,578–8,732)	−24 (−833–7,465)	79 (−950–8,201)	73 (−506–2,066)	143 (−1,920–6,079)	0.23
Δ PU 1 year after Bx	−143 (−5,781–3,099)	40 (−1,127–753)	117 (−3,619–1,577)	−115 (−405–2,005)	96 (−2,184–7,430)	0.12
Δ PU 3 years after Bx	132 (−5,308–2,434)	862 (−288–3,420)	−420 (−3,000–2,191)	220 (−184–525)	6 (−1,713–3,211)	0.22
Δ PU 5 years after Bx	146 (−2,606–2,429)	885 (−47–1,129)	637 (−1,566–1,232)	116 (−1,087–2,343)	427 (−653–1,891)	0.97

*The values were expressed as median and IQR*.

*AHT, antihumoral therapy; MFI, mean fluorescent intensity; eGFR, estimated glomerular filtration rate; ΔeGFR, difference of eGFR value; PU, daily urine protein excretion; ΔPU, difference of PU value*.

*Model A, the eGFR values after graft loss or death were not imputed; Model B, the eGFR values after graft loss or death were imputed as 5 ml/min/1.73 m^2^*.

** *p < 0.01, comparing with iTG;*

* *p < 0.05, comparing with iTG*.

Δ *p < 0.05, comparing with cAMR free of AHT*.

*The bold values indicates all p-values less than 0.05*.

### Patient and Graft Outcomes

**Figure 3:**
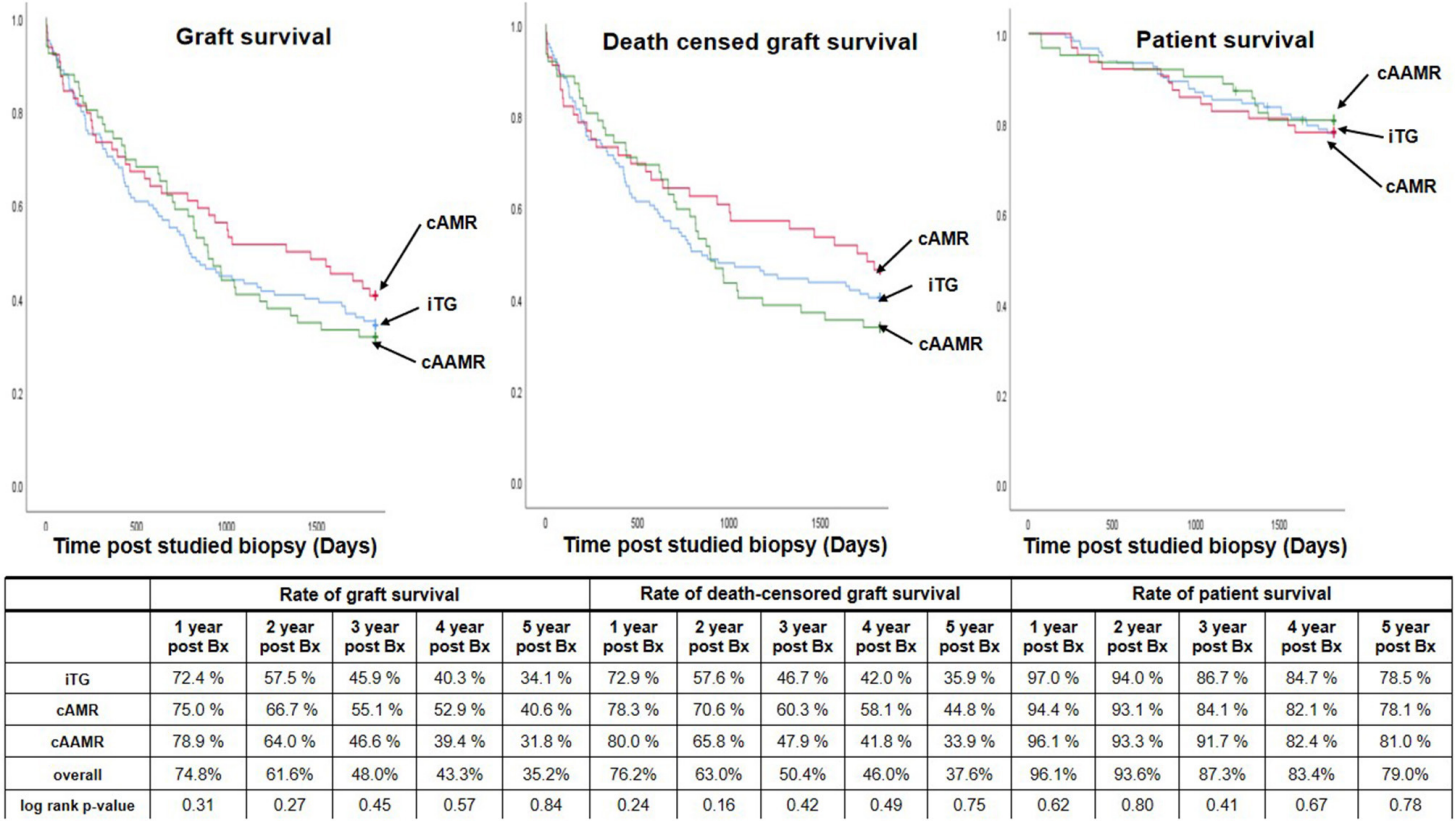
The comparison of 5-year graft survival **(left)**, death censored graft survival **(middle)** and 5-year patient survival **(right)** among iTG, cAMR and cAAMR groups. Bx, the studied biopsies.

**Figure 4:**
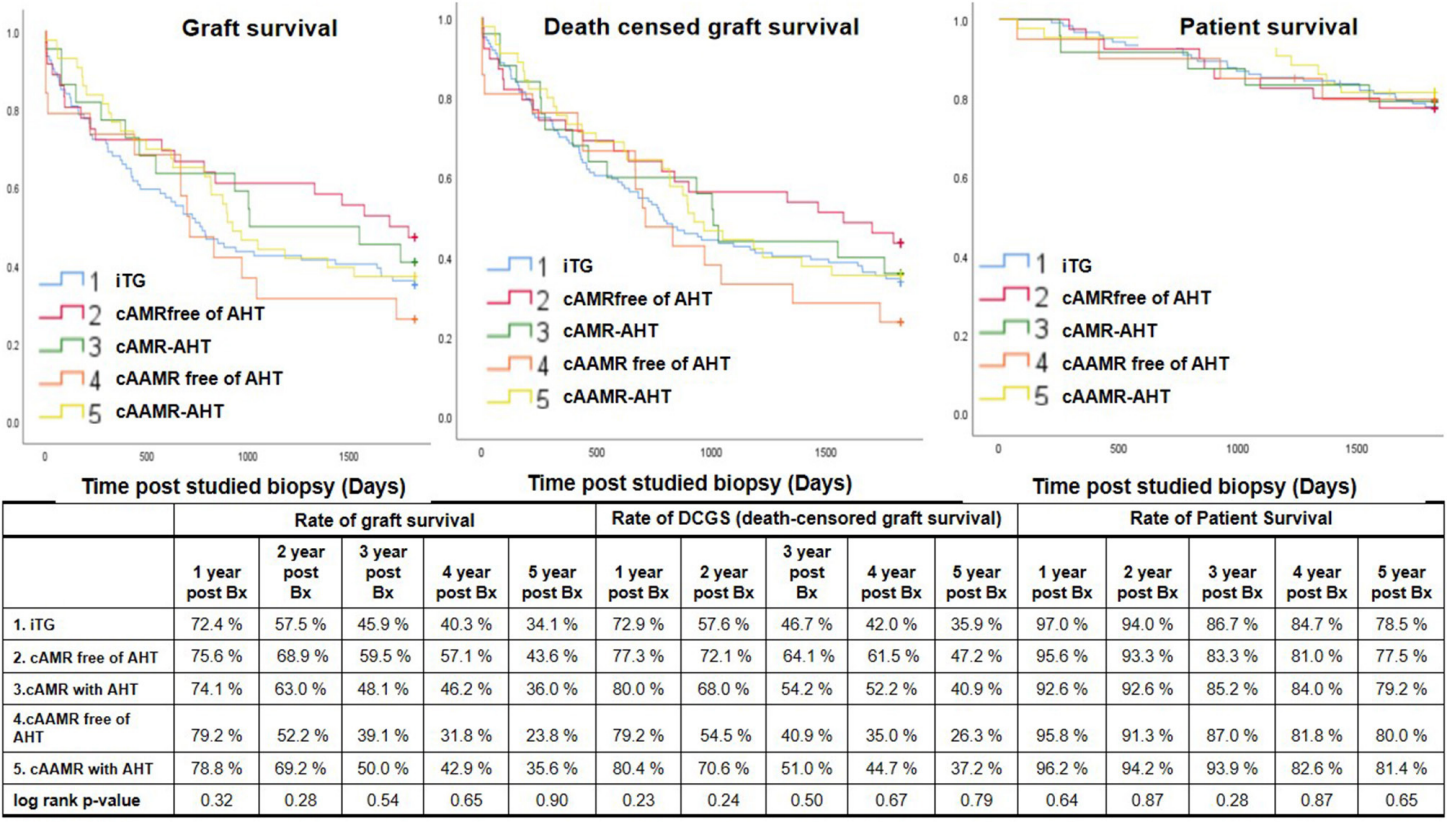
The effect of AHT on the 5-year post Bx graft survival rates **(left)**, DCGS rates **(middle)** and patient survival rates **(right)**. AHT, antihumoral therapy; Bx, the studied biopsies.

Importantly, the rates of graft survival (GS), death censored graft survival (DCGS) and patient survival (PS) at 1-, 3- and 5-year post transplantation were comparable between groups (Figures 3, 4). 5-year Kaplan-Meier estimate for DCGS after diagnosis of iTG, cAMR and cAAMR were 35.9, 44.8, and 33.9%, respectively (*P* = 0.75) and rates of GS, DCGS and PS were comparable among iTG, cAMR and cAAMR groups at each time during follow-up (Figure 3). Finally, 5-year Kaplan-Meier estimate for overall graft survival (including patient death) of iTG, cAMR and cAAMR were 34.1, 40.6, and 31.8%, respectively (*P* = 0.84).

The role of AHT on the long-term graft outcome is shown in Figure 4. Up to 5-year post studied biopsies, there were no significant differences of the GS, DCGS and PS rates upon comparison between the patients with or without AHT in cAMR and cAAMR group and similar in comparison with iTG group (*P* > 0.05).

During the 12 months after diagnosis, the episodes of urinary tract and respiratory tract infections that required hospitalization occurred with comparable frequency among iTG, cAMR and cAAMR groups.

### Correlation of Histological and Clinical Features With 5-Year Outcome

Each Banff lesion was divided into mild grade (score 0-1) and severe grade (score 2-3). After exclusion of thirteen patients, who died with a functioning graft, we found significant differences in 5-year death-censored graft survival when comparing mild and severe grade of Banff mm-, ah-, cv-, t-, ci- and ct-lesion in univariate Kaplan-Meier analysis (Supplementary Table 3), these six Banff lesions were consequently selected for further multivariable analysis. Based on clinical experience, we performed a binary-logistic regression to assess the association of three selected clinical variables (eGFR and proteinuria at biopsy, and receiving AHT) with 5-year postbiopsy graft survival, patient survival and death-censored graft survival. The Banff mm-, ah-, t-, ci- and ct-lesions as well as eGFR level at biopsy were closely associated with 5-year graft failure (Table 4).

**Table 4:** Binary logistic-regression analysis of clinical and histologicl factors associated with 5-year outcome after diagnosis of transplant glomerulopathy.

	**Graft loss**				**Patient death**				**Death-censored graft loss**			
	**OR**	**95% CI**		***p*** **-value**	**OR**	**95% CI**		***p*** **-value**	**OR**	**95% CI**		***p*** **-value**
**Clinical factors**											
eGFR value at Bx	0.97	0.95	0.99	**0.02**	0.96	0.92	0.99	0.05	0.97	0.95	0.99	**0.02**
PU value at Bx	1.00	1.00	1.01	0.10	1.00	1.00	100	0.43	1.00	1.00	1.01	0.06
Receiving antihumoral therapy	0.82	0.38	1.79	0.62	0.75	0.26	2.13	0.58	0.73	0.33	1.62	0.44
**Histological factors**									
mm >1	3.19	1.60	6.35	**0.001**	1.83	0.91	3.67	0.09	3.33	1.66	6.72	**0.001**
ci >1	2.39	1.26	4.55	**0.008**	1.49	0.77	2.89	0.24	2.56	1.33	4.91	**0.005**
ct >1	2.32	1.22	4.42	**0.01**	1.53	0.79	3.00	0.21	2.48	1.29	4.76	**0.006**
ah >1	2.79	1.24	6.27	**0.01**	1.34	0.46	3.87	0.59	2.77	1.20	6.40	**0.02**
t >1	2.98	1.05	8.48	**0.04**	1.13	0.39	3.25	0.83	3.02	1.04	8.76	**0.04**
cv >1	1.70	0.90	3.21	0.11	1.46	0.66	3.24	0.36	1.44	0.75	2.77	0.28

*eGFR, estimated glomerular filtration rate; PU, daily urine protein excretion*.

*OR, odds ratio; CI, conference intervals for odds ratio*.

*The bold values indicates all p-values less than 0.05*.

## Discussion

Late graft failure is a common problem after kidney transplantation presenting a seriously debilitating and life-threatening condition (35); AMR is considered as the major cause of late allograft loss outside of death with functioning graft and TG is recognized as a key histological change of chronic antibody-mediated injury during late allograft dysfunction (8). There is a need for robust surrogate endpoints in transplantation, that adequately predict long-term graft outcome and facilitates the performance of clinical trials (36). So far, several biomarkers have been considered as proposed endpoints for kidney allograft dysfunction (37) but there is rather limited experience of these surrogate endpoints on graft outcome. Only a few studies have sufficient numbers, long-term follow-up and are fulfilling the most recent diagnostic criteria for AMR (1, 38, 39). In the Banff 2017 report (12) the diagnosis of cAMR is well-defined and differentiated from the cAAMR. However, there is still significant ambiguity and knowledge gaps for the different histopathologic forms of TG (40). In this respective analysis, 282 patients developing TG after transplantation were investigated. using the strict, most recent Banff criteria and individual features (12), patients with TG were devided into iTG, cAMR and cAAMR categories, and the evolution of allograft function and long-term graft outcomes analyzed. Our analysis showed no significant differences in eGFR decline, proteinuria, DSA intensity and morphologic features among iTG, cAMR and cAAMR groups; moreover, no obvious benefit of AHT was found in treating patients of cAMR or cAAMR groups because on average more than 60% patients lost the allograft function within 5-year postbiopsy follow-up.

The development of TG is viewed as a structural ‘end-product’ of the antibody-mediated pathophysiological process (41), however, the quality and quantity (titer) of circulating DSAs may impact the clinical manifestation of the AMR (42, 43), and discrepancies between histological and serological findings are commonly exist (44). In this study, the patients with cAMR had lower DSA intensity, less C4d positivity and less frequent combined class I and II DSAs compared to cAAMR group. Previous studies showed that patients with exclusively weak or no complement-activating DSAs tended to experience less disease activity and eventually had better outcomes (45). Our data provide further evidence for a fluctuating activity and/or patchy distribution of AMR activity in the kidney, supporting the hypothesis that cAMR and cAAMR are a spectrum of the same disease due to a shared underlying pathophysiology.

Nearly all therapeutic approaches for treating AMR aim to remove circulating DSAs and to decrease DSA production (46) in order to reduce of DSA intensity and AMR-activity. However, irrespective of AHT, the cAMR and cAAMR patients had some longitudinal variation of DSA-MFI values without significant intergroup differences. Although IVIG/PPh is regarded as the “standard care of AMR” (47, 48), the DSA-producing plasma cells are not affected (49). In an attempt to prevent further antibody production, some patients received additional rituximab or bortezomib therapy. A prospective, randomized study (23) reported that treatment of late AMR with rituximab in combination with steroids IVIG and PPh did not improve any outcome parameter compared to placebo (23). Similarly a randomized trial did not show any therapeutic efficacy for bortezomib (50). The current evidence is in line with our data and supports that there is no proven treatment for cAMR and cAAMR (19).

This is one of the first studies to report a large cohort of iTG according to Banff 2017 criteria (12). TG is a frequent histological finding and could be a sign of AMR, but there is evidence that many TG cases do not have detectable DSA nor evidence for antibody interaction with graft vascular endothelium (51). A retrospective analysis of TG in 954 kidney transplant recipients (3,744 biopsies including protocol biopsies) observed TG in 10% of patients independent of HLA mismatches, and >75% of TG cases had no HLA-DSA. They concluded that iTG represents a different phenotype that had lower levels of concomitant inflammation and graft loss compared with HLA-DSA+ TG (52). In our study iTG was observed in 47.5% of indication biopsies without signs for AMR, and we could not detect significant differences in outcomes among iTG, cAMR and cAAMR during a 5-year follow-up. HLA-DSA negative TG may also be caused by antibodies against non-HLA targets including non-HLA antibodies (e.g., against minor histocompatibility antigens) or other targets such as endothelial antigens or vimentin (53) and the failure to demonstrate DSA in iTG cases does not rule out the contribution of other antibodies in the pathophysiology of TG (54). Alternatively, the absorption of low antibody levels by the allograft may result in a lack of circulating DSA (55). Until we have fully deciphered the pathophysiology we should consider iTG as a rather frequent separate disease category in the long-term course after transplantation, indicating structural damage of the glomerular basement as evidenced by proteinuria, and resulting in suboptimal outcomes.

Although TG is a heterogeneous condition, the underlying disease processes often share a final common clinical pathway of declining kidney graft function and increasing proteinuria (56). Several publications advocate the use of eGFR slope as a surrogate for clinical outcome in kidney disease trials (57, 58), although annualized GFR loss does not meet all criteria for a valid surrogate endpoint (59). In our study the three TG groups had a comparable annual eGFR decline and similar long-term outcomes without an effect of AHT. Also proteinuria is considered a potential useful biomarker which is associated with structural injury of glomerular basement membrane and a decline in kidney function (60). In our study, the urinary protein excretion was comparable among iTG, cAMR, and cAAMR groups but failed to reach statistical significance in the multivariable models for long-term outcomes.

Late graft failure often coincides with cumulative chronic histologic injury (61), which has previously been identified as strongly associated with allograft loss, irrespective of diagnosis (62). The biopsies performed in late period of transplantation are particularly dominated by non-specific chronic lesions and IFTA (63). Our biopsies with TG displayed moderate to severe transplant vasculopathy (by ah- and cv-lesions), which might further contribute to late graft loss (64). Although the median scores of ci- and ct-lesions in our patients with TG were not advanced, the presence of IFTA in combination with transplant vasculopathy might also indicate some potential CNI-nephrotoxicity. The long-term exposure to CNI has been proven as one of the major risk factors leading to arterial intimal fibroproliferation and neointimal thickening, eventually resulting in graft ischemia and striped IFTA (65) and predicting rather poor graft survival (66). In addition, the AHT regimen with enhanced immunosuppression led to a higher number of over immunosuppression and conferred a substantial risk of drug-toxicities, which was closely associated with the deterioration of the tubulointerstitial fibrosis and inferior late graft survival (67). Several studies highlight the importance of progressive fibrosis as a key pathway to graft failure and a target for intervention independent of the role of AMR in late graft failure (11, 62). Therefore, the ideal therapeutic guidelines for TG remain to be determined, and the choice of appropriate medication dosage, paired with careful patient monitoring and adjustment of baseline immunosuppression, needs to be investigated.

AMR is often initially detected with concomitant TCMR, and the treatment of concomitant TCMR is recommended in all cases of AMR (19, 68). We found significantly more concomitant TCMR in the cAAMR group than in the iTG and cAMR groups, in parallel with an evidently rapid decline in eGFR before studied biopsy. An additional steroid bolus was given to treat the mixed TCMR, and afterward the median eGFR decline at each time post studied biopsy between the cAMR and cAAMR groups, which might be explained by an adequate response of concomitant TCMR to steroids while the clincal course of AMR was not affected.

It is important to point toward the limitations of our study. First, the retrospective design of our study has inherent limitations and although all data were captured since 2000 in an electronic database, different biases are always present in retrospective data collections. Second, our results are obtained from indication biopsies and indication for biopsies may have changed over time. Our center does not perform protocol biopsies, which might have identified early subclinical lesions, which theoretically could better correlate with outcome than advanced lesions detected in indication biopsies. Third, TG is not per se a diagnosis, but a histologic lesion, which can be seen as a uniform response pattern of the glomerular basement membrane to different injuries, including AMR (18). Therefore it is difficult to completely exclude TMA of other causes or de novo/recurrent glomerulonephritis, which may have been misdiagnosed as TG in the absence of immune complexes. However, we relied on the most recent consensus from the Banff 2017 classification and it seems unlikely that such misdiagnoses have introduced a significant bias in this study.

In summary, our observational study demonstrates that the occurrence of TG is associated with poor long-term graft outcomes independent of the TG categories and scores. Therefore our data point toward the limitations of TG grading as a suitable potential surrogate endpoint for clinical trials. Given that late graft failure (excluding death) is often multifactorial (3), and TG may arise as a uniform “response to injury pattern” from different underlying diseases the isolated histopathological finding of TG as single surrogate endpoint may not fully reflect the complexity of graft loss in kidney transplantation and cg grading was not associated with outcome. Contrary, Banff scores associated with chronic scarring might be better suited to predict an unfavorable outcome in patients with TG. Importantly, AHT in patients with AMR had no relevant effect on the fluctuating course of DSA, eGFR decline and long-term allograft outcome. Our findings clearly support the need for prospective, randomized trials in this area. Meanwhile, when approaching the use of existing AHT agents for treating cAMR or cAAMR, less may be more.

## Data Availability Statement

The original contributions presented in the study are included in the article/Supplementary Material, further inquiries can be directed to the corresponding author/s.

## Author Contributions

KW and BR participated in evaluation of pathologic slides, research design, and writing. DS participated in data administration. CL and NL participated in detection of DSA. BO, FH, MN, MC, FB, SR, WD, and ES participated in the designation and performance of the research. KB participated in the research design and paper writing. All authors have read and agreed to the published version of the manuscript.

## Funding

MN, ES, and WD are participants in the BIH Charité Digital Clinician Scientist Program funded by the Charité-Universitätsmedizin Berlin, the Berlin Institute of Health and the German Research Foundation (DFG).

## Conflict of Interest

The authors declare that the research was conducted in the absence of any commercial or financial relationships that could be construed as a potential conflict of interest.

## Publisher's Note

All claims expressed in this article are solely those of the authors and do not necessarily represent those of their affiliated organizations, or those of the publisher, the editors and the reviewers. Any product that may be evaluated in this article, or claim that may be made by its manufacturer, is not guaranteed or endorsed by the publisher.
