# Supplementary material for: Poor Outcomes in Patients With Transplant Glomerulopathy Independent of Banff Categorization or Therapeutic Interventions
Source: Front Med (Lausanne). 2022 May 12;9:889648. doi: 10.3389/fmed.2022.889648 (PMC9133540; doi:10.3389/fmed.2022.889648)
Supplement: Supplementary file 1 [file Table_1.DOC]

Supplementary TABLE S1| Assessing differences of eGFR slope among three TG categories by linear mixed models


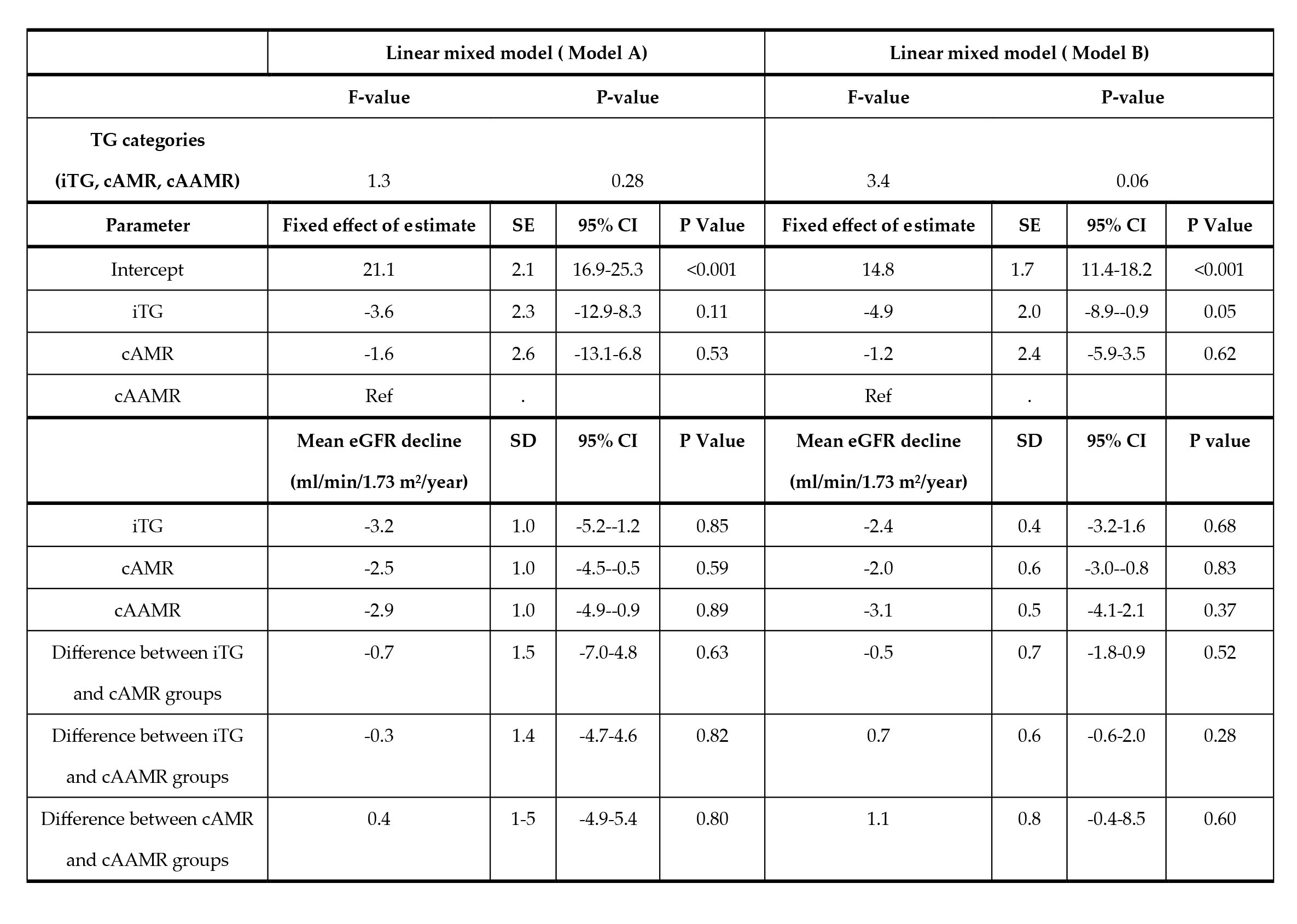


Linear mixed models fit by maximum likelihood t-tests use Satterthwaite approximations to degrees of freedom. The intercept corresponds to the (unweighted) grand mean. For each factor with k levels, k-1 parameters are estimated, consequently, the estimates cannot be directly mapped to factor levels.

SE: standard error; SD: standard deviation; CI: confidence interval; Ref: as reference for comparison;

iTG: isolated transplant glomerulopathy; cAMR: chronic antibody-mediated rejection; cAAMR: chronic active antibody-mediated rejection;

Model A: the eGFR values after graft loss or death were not imputed;

Model B: the eGFR values after graft loss were imputed as 5 ml/min/1.73 m^2^

Supplementary TABLE S2| Assessing differences of eGFR slope by linear mixed models in relation to AHT


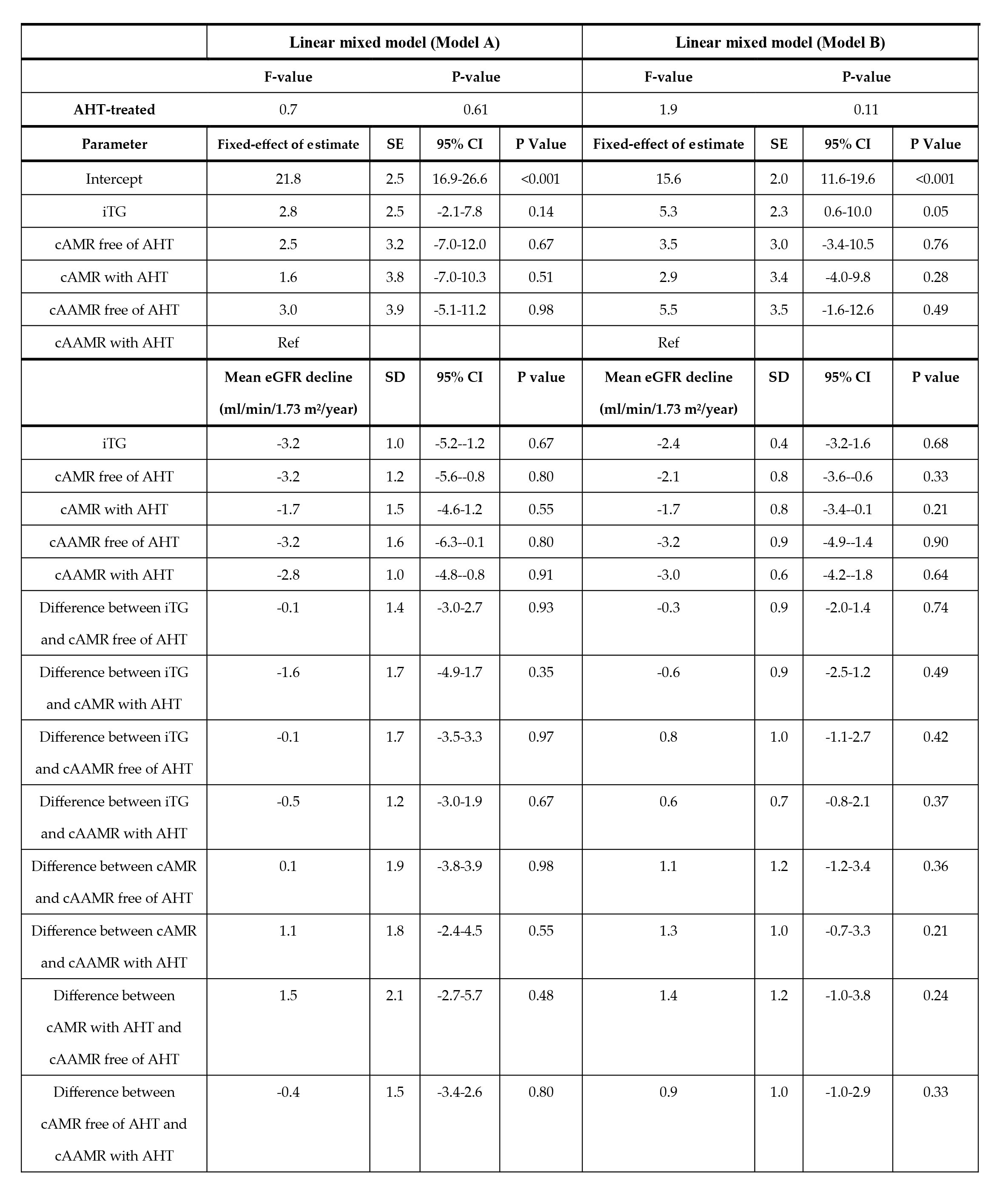


Linear mixed models fit by maximum likelihood t-tests use Satterthwaite approximations to degrees of freedom.

The intercept corresponds to the (unweighted) grand mean. For each factor with k levels, k-1 parameters are estimated, consequently, the estimates cannot be directly mapped to factor levels.

SE: standard error; SD: standard deviation; CI: confidence interval; Ref: as reference for comparison;

iTG: isolated transplant glomerulopathy; cAMR: chronic antibody-mediated rejection;

cAAMR: chronic active antibody-mediated rejection;

AHT: antihumoral therapy;

Model A: the eGFR values after graft loss or death were not imputed;

Model B: the eGFR values after graft loss were imputed as 5 ml/min/1.73 m^2^

Supplementary TABLE S3| Comparing 5-year death-censored graft survival between cases with mild and severe Banff lesions in univariate Kaplan-Meier analysis


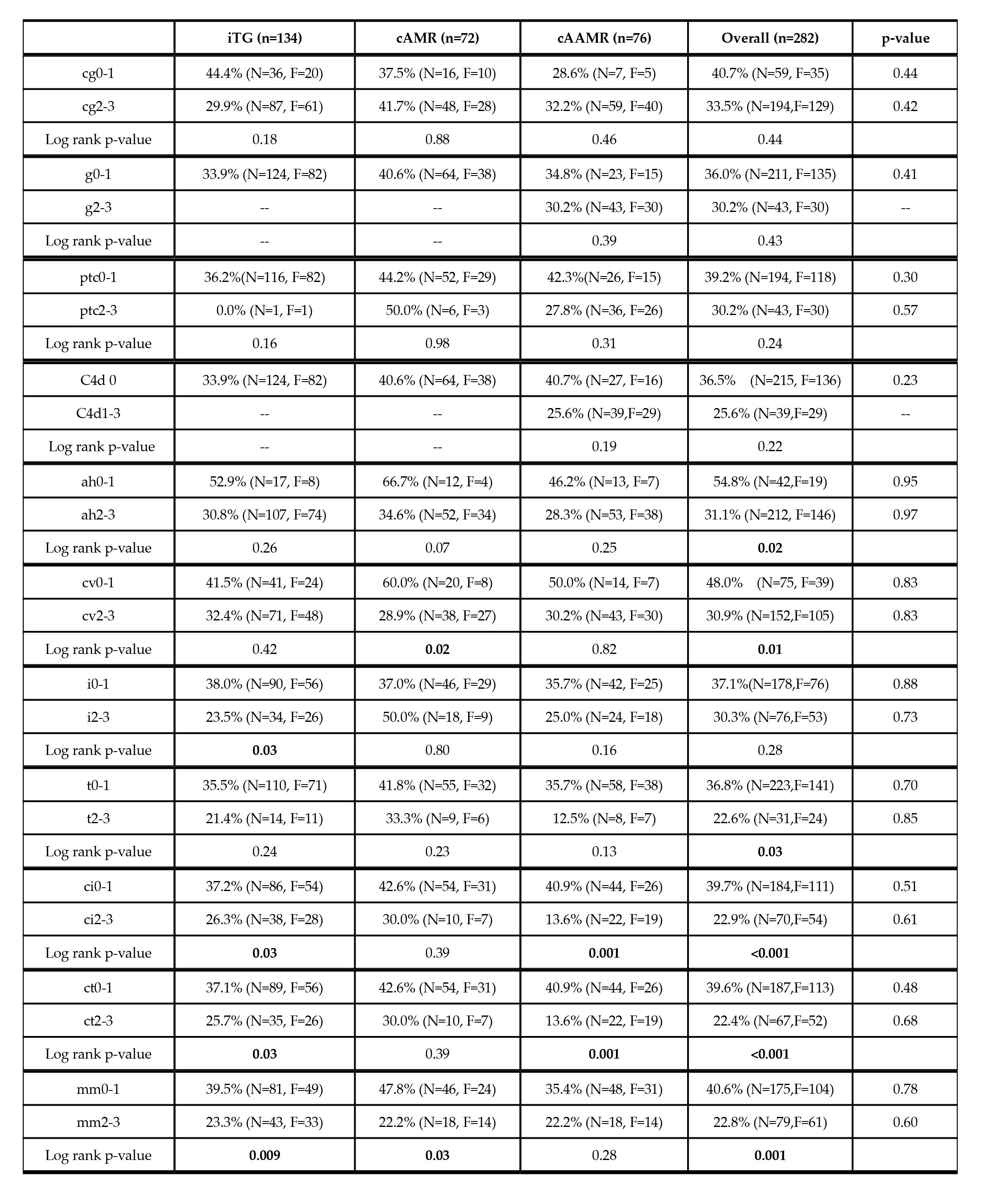


N: number of biopsies; F: number of graft failure
